# The Effect of Beta-Trace Protein on Diagnosis and Prognosis in Patients with Acute Coronary Syndrome

**DOI:** 10.7759/cureus.7135

**Published:** 2020-02-28

**Authors:** Ekrem T Sert, Nazire Akilli, Ramazan Köylü, Basar Cander, Kamil Kokulu, Öznur Köylü

**Affiliations:** 1 Emergency Medicine, Aksaray University Medical School, Aksaray, TUR; 2 Emergency Medicine, Konya Training and Research Hospital, University of Health Sciences, Konya, TUR; 3 Emergency Medicine, Kanuni Sultan Süleyman Training and Research Hospital, University of Health Sciences, Istanbul, TUR; 4 Emergency Medicine, University of Health Sciences, Ümraniye Training and Research Hospital, Istanbul, TUR; 5 Biochemistry, Konya Training and Research Hospital, University of Health Sciences, Konya, TUR

**Keywords:** acute coronary syndrome, beta-trace protein, atherosclerosis, markers, dıagnosıs

## Abstract

Objective

The purpose of this study was to determine the effect of beta-trace protein (BTP) levels at the time of admission and at 8th hour on diagnosis and prognosis in patients who were under treatment and follow-up with acute coronary syndrome (ACS) diagnosis at coronary intensive care unit and emergency department.

Materials and Methods

This study was conducted between June 2014 and December 2014 at the Emergency Department of Konya Training and Research Hospital. Demographic characteristics, background, vital findings, laboratory findings, blood BTP levels, coronary angiography results, and echocardiography findings of the patients diagnosed with ACS were recorded. Risk classification was performed for patients with ACS and their mortality rates were recorded. Relation of BTP level with risk classification and mortality was evaluated.

Results

A total of 174 individuals, 138 patients and 36 control subjects, were included in the study. No significant difference was detected between BTP levels at the time of admission and at 8^th^ hour in the patient group (p=0.883). There was no difference between the patient and control groups in terms of the BTP level (p=0.335). Ten patients (7.2%) died in the patient group. BTP levels measured at the time of admission and at 8th hour were not different for dead and living patients (admission p=0.085, 8^th^ hour p=0.141).

Conclusion

We determined that there was a lack of biochemical markers that could be used for the prognosis of serum BTP levels in patients admitting to the emergency unit with ACS.

## Introduction

Beta-trace protein (BTP), also known as prostaglandin D synthase (PGDS), is a protein with a low molecular weight that belongs to the lipocalin protein family. PGDS catalyzes the isomerization of Prostaglandin(PG) H2, a common precursor of various prostanoids, to PGD2(1). There are two types of PGDS, which have been derived from phylogenetically different protein families [1]. The first is hematopoietic PGDS, which belongs to the glutathione (GSH)-S-transferase family and requires glutathione for its function [2]. The second is lipocalin-type PGDS, which is a glutathione-independent enzyme, also known as BTP [3].

Recently, BTP has been detected in the heart in myocardial cells, atrial endocardial cells, intimal smooth muscle cells, and in fibrous plaques of coronary arteries with atherosclerosis (excluding normal coronary arteries) [4-5]. In various studies involving human and animal models, BTP has been demonstrated to play a protective role in the heart under hypoxia and ischemia [6]. It also has a biological role in vasodilatation, bronchoconstriction, thrombocyte aggregation inhibition, and inflammatory cell processes [7]. These findings suggest that BTP may play a role in the pathophysiology of cardiovascular diseases. It may thus have a prognostic value. In the present study, we aimed to determine the effect of serum BTP levels on diagnosis and prognosis in patients presenting to the emergency service with acute coronary syndrome (ACS).

## Materials and methods

Subjects and study design

Patients aged over 18 years who presented to the Emergency Service of Konya Training and Research Hospital between June 2014 and December 2014 were evaluated. In total, 150 patients who were diagnosed with ACS and 40 volunteers were planned to be included in the study. Twelve patients and four individuals from the control group were excluded because of hemolysis in their BTP blood test results. A total of 138 patients and 36 healthy volunteers were finally included in the study. Local ethics committee approval (2014 /82) was obtained before commencing the study.

Demographic characteristics, medical histories, vital signs, laboratory findings, coronary angiography (CAG) findings, and echocardiography (ECG) findings of the patients were recorded. According to their CAG results, the patients were classified into three groups: one arterial lesion, two arterial lesions, and three arterial lesions. Patients with ≥50% stenosis according to CAG results were considered as having a significant coronary artery disease (CAD), and those with ≤50% stenosis were regarded as having non-critical stenosis.

To assess the clinical risk factors, all patients were divided into two groups: patients with ACS with ST elevation (STEMI) and without ST elevation (NSTEMI, unstable angina pectoris [UAP]). Requirements for STEMI classification were as follows: a) Typical chest pain and/or ischemic symptoms during rest lasting more than 20 min; b) In ECG, in two consecutive derivations, starting from point J, an ST elevation of ≥0.1 mV in all derivations other than V2-V3, an ST elevation of ≥0.2 mV at V2-V3, and a change in ST-T that is considered to be recent or a recent left bundle branch block; and c) positive markers for myocardial necrosis. Requirements for NSTEMI classification were as follows: a) as described previously, absence of ST segment elevation and b) positive markers for myocardial necrosis. Requirements for UAP classification were as follows: a) as described previously, absence of ST segment elevation of ≥1 mm and b) negative markers for myocardial necrosis and presence of angina pectoris in any of the three following cases: angina that occurs at rest or for a prolonged period of time (usually >20 min), new-onset angina of at least class III severity according to the classification of the Canadian Cardiovascular Society (CCS) or recent progression of at least one CCS class in pain classification, or a new angina progression of at least CCS class III [8].

While the clinical risk assessment of patients without ST elevation (NSTEMI and UAP) was conducted using the Global Registry of Acute Coronary Events (GRACE) risk scoring, STEMI Thrombolysis in Myocardial Ischemia (TIMI) risk scoring was used in STEMI patients. In the GRACE scoring system, risk scores of age, heart rate, systolic blood pressure, Killip class, ECG, and cardiac marker changes, creatinine level, and cardiac arrest history variables are calculated using computer programs, and the patients are categorized into three risk groups according to these scores: low-risk, medium-risk, and high-risk groups. These scores have significant discriminative power in the assessment of the risk of death at the hospital, discharge from hospital, and 6-month mortality risks of patients [9, 10]. In STEMI, the TIMI risk score is an important tool in evaluating the mortality risk. Age, weight, duration of treatment, heart rate, systolic blood pressure, Killip class, ECG changes, DM or HT or angina history are assessed, and patients are categorized into two groups: low-risk and high-risk groups [11].

Patients with a known renal disease or those who had received renal replacement therapy; pregnant or breastfeeding patients; patients with an inflammatory disease, connective tissue disorder, hepatic impairment, or infection; patients who had myocardial infarction or had been diagnosed with pulmonary embolism in the previous 3 months; unconscious patients; patients aged <18 years; and those who did not give consent to participate in the study were excluded from the study. Individuals who presented to the green zone of the hospital’s emergency service and were not diagnosed with any disease as a result of the examinations did not have a chronic disease, were aged >18 years and who gave consent were included in the control group. Blood samples were collected from the patients within the first 15 min after they were diagnosed with ACS at the emergency service. Three cubic centimeters of blood were taken into a biochemistry tube, and the BTP level at admission was analyzed. Another blood sample was taken from all participants 8 hours after admission to the emergency department, and the BTP level was analyzed again. The treatment, duration of hospitalization, and hospital mortality rates were monitored.

Measurement of serum BTP level

Venous blood samples were collected from all patients included in the study. Samples were put into BD Vacutainer® SST™ II Advance Serum Separator tubes. The blood in the tubes was centrifuged using a Hettich Rotina 38R centrifuge device at 4000 rpm for 7 min and stored at −80 C. Serum and plasma were thawed at the same time at room temperature. Serum BTP levels were analyzed. Serum BTP concentration was measured using time-resolved fluorescence immunoassay based on the components of a commercially available enzymatic immunoassay kit (Cayman Chemical, Ann Arbor, MI, USA). During the analyses, a BIO-TEK model ELX-50 was used as a microplate washer. Results were recorded using a BIO-TEK model ELX800 microplate reader. All parameters were read at 450 nm, and the absorbance values obtained from the device were placed in the calibration graph to obtain sample results in mcg/dl.

Statistical analysis

Descriptive analyses were expressed as the mean ± standard deviation for normally distributed variables and median and interquartile range (IQR) for non-normally distributed variables; categorical data were expressed as n (%). When comparing data between the ACS group and the control group, chi-square or Fischer’s exact tests were used for categorical data and independent samples t-test was used for normally distributed variables. Non-normally distributed variables were compared using the Mann-Whitney U test. BTP levels of patients at admission and at 8 h and other laboratory parameters were compared using the t-test for normally distributed variables and using the Wilcoxon test for non-normally distributed variables. The patient group was classified into three sub-groups according to their angiography results: those with one arterial lesion, two arterial lesions, and three arterial lesions. Intergroup differences were assessed using the Kruskal-Wallis test for numerical data and chi-square or Fischer’s exact test for categorical data. A P-value below 0.05 was considered statistically significant.

## Results

The mean age of 138 patients included in the study was 66.4 ± 14.0 years, and the mean age of the control group was 52.5 ± 14.6 years. Of the patients, 47 were women and 91 were men (34.1% vs. 65.9%). Further, 69 patients were assessed as NSTEMI and 69 as STEMI (50% vs. 50%). Demographic characteristics of the patients are shown in Table 1.

The comparison of blood BTP levels of the patients between admission and at 8 h did not reveal a significant difference (p = 0.883). When the study and control groups were compared, there was no statistically significant difference between the BTP levels at admission (p = 0.335) (Table 2).

**Table 1:** Clinical characteristics of the participants Data are expressed as mean ± standard deviation (SD), as number (percentage), or as median (IQR); NSTEMI, non-ST segment elevation myocardial infarction; STEMI, ST-segment elevation myocardial infarction; BTP, beta-trace protein.

Variable	Study Population (n = 138)
Age (years) (mean ±SD)	66,4±14,0
Gender (%)	
Male	91(65,9%)
Female	47(34,1%)
Systolic blood pressure (mm Hg)	122±23,5
Diastolic blood pressure(mm Hg)	73,2±12,7
Heart rate (beats/min)	81,1±16
Glucose (mg/dL)	113,5(79,2)
Diabetes mellitus	46 (33,3%)
Hypertension	83 (60,1%)
Hyperlipidemia	42(30,4%)
Coronary artery disease	41(29,7%)
Habitual smoking	49(35,5%)
Family history	39(28,3%)
Admission BTP (microg/dl)	589,6 (315,9)
Hour 8 BTP (microg/dl)	619,5 (329,1)
Admission creatinine (mg/dL)	0,9(0,3)
Admission urea (mg/dL)	38,6±13,2
STEMI	69(50%)
NSTEMI	69(50%)
Ejection fraction	50±12,5
Left ventricular dysfunction	106(76,8%)
Coronary angiography	
1-vessel disease	60 (%43,4)
2-vessel disease	21 (%15,2)
3-vessel disease	34 (%24,6)
30-Day mortality	10(7,2%)

**Table 2:** Study/control group and BTP levels BTP, beta-trace protein; SD, standard deviation; IQR, ınterquartile range

	Study Population (n = 138)	Control Group (n = 36)	P-value
Gender (%) Male	91(65,9%)	15(41,6%)	0,008
Age (years) (mean ±SD)	66,4±14,0	52,5±14,6	0,882
BTP microg/dl median (IQR)	597,1(314,8)	542,8(346,8)	0,335

The patients were divided into two groups: low ejection fraction (EF) group, with EF <40%; and high EF group, with EF >40%. Thirty-three (23.9%) patients had low EF. When EF values of patients and their BTP levels at admission and at 8 h were compared, no statistically significant relationship was found (p > 0.05).

There was no significant difference in BTP levels at admission and at 8 h between the NSTEMI and STEMI groups (p > 0.05). Following CAG, stenosis was detected in three coronary arteries in 34 (24.6%) patients, in two coronary arteries in 21 (15.2%) patients, and in one coronary artery in 60 (43.4%) patients. There was no significant difference between the BTP levels at admission and at 8 h among these groups (p > 0.05).

BTP levels of low-, medium-, and high-risk groups according to the GRACE risk score were compared. No significant difference was found between the BTP levels at presentation and at 8 h among these groups (p > 0.05). In STEMI TIMI, the BTP level at admission in the high-risk patient group was found to be higher, which was close to statistical significance (p = 0.058) (Table 3). Ten of the 138 patients died. The groups with and without mortality are compared in Table 4. Comparison of BTP levels among these two groups did not reveal a significant difference at admission and at 8 h (BTP at admission, p = 0.085; BTP at 8 h, p = 0.141). When the groups with and without mortality were compared, EF (p = 0.013), creatinine at admission (p = 0.014), and GRACE risk score (p = 0.013) were found to be statistically significant.

Sixty-nine patients were evaluated as NSTEMI and 69 as STEMI (50% vs. 50%). No significant difference was found between these two groups in terms of BTP levels measured at the time of admission and at 8th hour (p > 0.05). CAG results showed that 34 (24.6%) patients had narrowing in three coronary vessels, 21 (15.2%) had in two and 60 (43.4%) had narrowing in one coronary vessel. Three groups were formed based on the number of vessel with narrowing. There was no significant difference between BTP levels measured at the time of admission and at 8th for these groups (p > 0.05).

Low-, moderate-, and high-risk groups of GRACE risk score and BTP levels were compared. No significant difference was found between three groups based on GRACE risk score in terms of BTP levels measured at admission and at 8th hour (p > 0.05). In high-risk patients with STEMI TIMI, BTP levels obtained at the time of admission were found to be higher, close to the level of significance (p = 0.058) (Table 3). Ten out of 138 patients died. Comparison of groups with and without mortality developed is presented in Table 4. Comparison of BTP levels between these two groups revealed no significant difference in terms of BTP values measured at the time of admission and at 8th hour (admission BTP p = 0.085, 8th hour BTP p = 0.141). Comparison of groups with and without mortality developed revealed statistically significant EF (p = 0.013), admission creatinine (p = 0.014) and GRACE risk score (p = 0.013).

 

**Table 3:** Comparison of risk groups and BTP level Data are expressed as median (IQR); BTP, beta-trace protein; STEMI, ST-elevation myocardial infarction; TIMI, the thrombolysis in myocardial ınfarction; GRACE, Global Registry of Acute Coronary Events; IQR, interquartile range

Variable				P value
STEMI TIMI score	Low risk	High risk		
Admission BTP level	545,9(273,9)	664,7(381,9)		0,058
Hour 8 BTP level	562,9(397)	632,3(336,7)		0,473
GRACE risk score	Low risk	İntermediate risk	High risk	
Admission BTP level	584,1(416,0)	671,9(262,6)	733,4(376,9)	0,455
Hour 8 BTP level	570,8(336,5)	621,5(310,7)	638,2(354,7)	0,585
Coronary angiography	1-vessel disease	2-vessel disease	3 vessel disease	
Admission BTP level	606,4(378,6)	623,4 (276,3)	564,1(550,4)	0,913
Hour 8 BTP level	616,6(379,3)	527,2(308,1)	697,5(365,3)	0,249

**Table 4:** Characteristics of living and dying groups Data given as median (quartiles), mean ± SD or n (%); BTP, beta-trace protein;  IQR, interquartile range

Variable	Live n: 128	Death n:10	P value
Age (years) (mean ±SD)	66±13,1	73,7±23,2	0,330
Gender (%) Male	85(66,4)	6(60)	
Admission BTP (microg/dl)	590 (292,8)	740,6 (874,8)	0,085

## Discussion

According to the results of the present study, serum BTP level cannot be used as a biochemical marker in the diagnosis and estimation of prognosis in patients with ACS.

CAD is currently one of the leading causes of death in industrialized countries. It has been found that over 1.5 million people have acute myocardial infarction annually in the United States of America [12]. In patients with ACS, biochemical markers that indicate myocardial damage play a vital role in both diagnosing the disease and in guiding the treatment. In recent years, the BTP level has been reported to be associated with CAD. BTP is suggested as a potential new diagnostic tool in stable CAD. In a study by Sergio Manzano-Fernandez *et al*. to assess the importance of increased plasma BTP for risk classification of patients with NSTEMI, the patients who died had higher levels of BTP, cystatin C (CysC), and serum creatinine and a lower estimated glomerular filtration rate. BTP and CysC were found to be determinants of all-cause mortality [13]. Inoue T et al. demonstrated that higher serum BTP levels led to an increased number of affected arteries in patients with stable angina. They suggested that BTP measurements may be used to diagnose patients with stable CAD and assess the severity of disease [14]. In their study, Miwa et al. studied the relationship between BTP level and presence of subclinical atherosclerosis in asymptomatic patients. Increased serum BTP level was shown to be associated with the progression of atherosclerosis. In addition, BTP levels were found to be associated with age and hypertension [15]. We could not verify these relationships in the present study.

Because BTP cannot be removed during hemodialysis, it has been used as an endogenous filtration marker in the evaluation of renal function [7]. There are studies showing that BTP may be used as a useful marker that can be measured in blood and urine for determining the severity of renal damage in chronic renal impairment, as an alternative to alpha 1-microglobulin and albumin in determining tubular damage [16]. Another study suggests that cystatin C was the most suitable marker in patients without tubular damage who underwent renal transplantation [17]. In a study conducted to evaluate the relationship between BTP and cardiovascular side effects, mortality, and hemorrhage in patients with AF, BTP levels and GFR were found to be negatively correlated. High BTP levels were found to be significantly correlated with the development of cardiovascular side effects and mortality in embolic events [18].

In the present study, no difference could be found between BTP levels at admission and at 8 h in either group and in BTP levels at admission between the patient and control groups. Patients with a known renal disease were not included in the study. These results demonstrate that the increase in BTP level is associated with renal failure [7,17-18]. This difference in our results can be attributed to the exclusion of patients with known renal disease. The aforementioned studies show that BTP has potentially superior attributes for evaluating renal functions. BTP acts as an indicator of biological and pathological processes for therapeutic intervention or pharmacological reactions and may also be used in diagnostic procedures of renal pathologies. Contrary to previous analyses, which found a relationship between BTP and CAD [13,14,19-20], our data showed that the BTP level is not high in patients with ACS with preserved renal function.

The low number of patients is the major limitation of the present study. The effectiveness of only one biomarker could be evaluated. We believe that other biomarkers that may be valuable for diagnosis and prognosis should also be used in conjunction with BTP.

## Conclusions

In the literature, many markers have been used to determine prognosis and mortality in ACS, and there are numerous risk scoring systems. Considering the findings from the present study and previous studies, we determined that serum BTP level is not suitable biochemical marker for the diagnosis and estimation of prognosis in patients with ACS presenting to the emergency department. We think that the relationship between BTP level and prognosis found in previous studies on ACS is because of the inclusion of patients with renal impairment in these studies. We believe that serum BTP level is not a biochemical marker that can be used for early-stage risk assessment in patients with normal renal function.

## Appendices

Disclosures

Ethical approval: Necmettin Erbakan University Meram Faculty of Medicine Scientific Research Evaluation Committee approval was obtained for this study (approval number: 2014 /82). Human rights: The study protocol conforms to the ethical guidelines of the 1975 Declaration of Helsinki. Declaration of conflicting interests: The author(s) declared no potential conflicts of interest with respect to the research, authorship, and/or publication of this article. Funding: The author(s) received no financial support for the research, authorship, and/or publication of this article.
